# Differential Serum MicroRNA Profiling in Benign and Malignant Ovarian Tumours: An Exploratory Study Identifying hsa-miR-200c-3p as a Candidate Diagnostic Biomarker

**DOI:** 10.3390/diagnostics16172718

**Published:** 2026-08-26

**Authors:** Jose D. Santotoribio, Juan Corral-Perez, Laura Avila-Cabeza-de-Vaca, Manuel Costilla, María Mayor-Reyes, Daniel Fatela-Cantillo, Jose M. Silvan, Cristina Casals, Jesus G. Ponce-Gonzalez, Pablo Mesa-Suarez, Carmen González Macías, Juan Jesús Fernández Alba

**Affiliations:** 1 Department of Laboratory Medicine, Puerto Real University Hospital, 11510 Cadiz, Spain; josed.santotoribio.sspa@juntadeandalucia.es (J.D.S.); maria.mayor.sspa@juntadeandalucia.es (M.M.-R.); 2 Institute for Biomedical Research and Innovation of Cádiz (INiBICA), 11003 Cadiz, Spain; juan.corral@inibica.es (J.C.-P.); laura.avila@gm.uca.es (L.A.-C.-d.-V.); manueljesus.costilla@gm.uca.es (M.C.); cristina.casals@uca.es (C.C.); jesusgustavo.ponce@gm.uca.es (J.G.P.-G.); mesa.pms@gmail.com (P.M.-S.); 3 ExPhy Research Group, Department of Physical Education, University of Cádiz, 11519 Cádiz, Spain; 4 Department of Laboratory Medicine, Virgen del Rocío University Hospital, 41013 Seville, Spain; daniel.fatela.sspa@juntadeandalucia.es; 5 Department of Obstetrics and Gynecology, Virgen del Rocío University Hospital, 41013 Seville, Spain; josem.silvan.sspa@juntadeandalucia.es; 6 Department of Anesthesiology, Resuscitation and Pain Treatment, Puerto Real University Hospital, 11510 Cadiz, Spain; 7 Department of Obstetrics and Gynecology, Puerto Real University Hospital, 11510 Cadiz, Spain; 8 Department of Maternal and Child Health and Radiology, University of Cádiz, 11009 Cadiz, Spain

**Keywords:** circulating microRNA, hsa-miR-200c-3p, ovarian carcinoma, liquid biopsy, diagnostic biomarker, adnexal mass

## Abstract

**Background/Objectives:** Preoperative discrimination of benign from malignant adnexal masses remains a major clinical challenge, and circulating microRNAs (miRNAs) are candidate non-invasive biomarkers. This exploratory, hypothesis-generating study—not a diagnostic validation study—evaluated serum hsa-miR-200c-3p as a candidate diagnostic biomarker for ovarian malignancy. **Methods:** Serum from 39 women with an adnexal mass and a surgical indication (20 benign, 19 malignant), all with histological confirmation, was analysed. Of 179 assayed miRNAs, 178 were tested for differential expression (miR-103a-3p as endogenous reference) using the Mann–Whitney U test with Benjamini–Hochberg false-discovery-rate (FDR) correction; hsa-miR-200c-3p, a pre-specified miR-200 family candidate, was evaluated by ROC analysis and compared with, and adjusted for, CA 125, HE4 and age. **Results:** The malignant group was substantially older and more frequently postmenopausal. Thirty-eight miRNAs were significant after FDR correction; hsa-miR-200c-3p was up-regulated in malignancy. In the complete-case analysis restricted to the 35 participants with a detectable marker (20 benign, 15 malignant), the AUC was 0.893 (95% CI 0.76–0.99); however, four high-grade serous carcinomas showed undetectable serum hsa-miR-200c-3p, and when these were included and imputed as low expression (all-case analysis, n = 39) the AUC fell to 0.705 (95% CI 0.51–0.88). hsa-miR-200c-3p did not demonstrate statistically superior performance to HE4 (AUC 0.93) or CA 125 (0.89), with overlapping confidence intervals; any incremental value beyond established markers and age was modest and exploratory. **Conclusions:** Serum hsa-miR-200c-3p is a preliminary candidate that requires prospective, age-matched, adequately powered validation with pre-specified assays and analysis plans.

## 1. Introduction

Ovarian cancer (OC) remains the most lethal gynaecological malignancy worldwide, characterised by late-stage diagnosis and high chemoresistance. Its poor prognosis is primarily due to asymptomatic progression in early stages, high rates of chemoresistance and metastasis, and the failure of current screening methods to detect localised disease. Current diagnostic standards, including transvaginal ultrasonography and serum tumour markers, show insufficient specificity for accurately distinguishing benign from malignant ovarian tumours, leading to unnecessary surgery for benign lesions and delayed referral of patients with malignancy to specialised centres [1,2,3,4]. Consequently, there is a need for specific biomarkers that complement current imaging and improve patient stratification.

MicroRNAs (miRNAs), a class of small non-coding RNAs of approximately 22 nucleotides, have emerged as potent modulators of OC [3,4,5]. Through post-transcriptional regulation of gene expression, miRNAs govern critical oncogenic processes including proliferation, apoptosis and chemosensitivity [6,7]. Profiling studies indicate that high-grade serous ovarian carcinoma exhibits a distinct miRNA signature compared with benign tissue. Previous investigations established the up-regulation of miR-21, miR-100 and members of the miR-200 cluster in malignant phenotypes, with expression levels associated with disease progression and metastatic dissemination [7,8,9]. The miR-200 family, in particular, is consistently up-regulated in malignant tissues, showing robust associations with disease progression, metastatic dissemination and therapeutic resistance [3,7,8,10].

Beyond tissue-specific roles, miRNAs are stable in the systemic circulation, protected from endogenous RNase activity by extracellular vesicles or protein conjugation [2,5,11], which supports their use as non-invasive biomarkers [4,6,8,12]. However, the clinical utility of circulating miRNAs has been limited by poor reproducibility and reliance on single-marker univariate analyses [3,4]. Robust biomarker development requires rigorous statistical evaluation with adequate control of multiple testing and confounding [7,11,13,14].

In this study we evaluated the serum expression of 179 miRNAs in patients with benign adnexal masses and ovarian carcinoma. Rather than relying on unstable data-driven feature selection in a small sample, we pre-specified hsa-miR-200c-3p—a well-characterised member of the miR-200 family—as the primary candidate biomarker, and evaluated its diagnostic performance with formal sensitivity analyses and comparison with established markers (CA 125 and HE4). Multivariable analyses (PCA and LASSO regression) were used as supporting, exploratory evidence.

## 2. Materials and Methods

This exploratory, cross-sectional diagnostic-accuracy study included 39 participants consecutively recruited within the project “Diagnostic algorithms using biomarkers identified in blood and urine for the early diagnosis of ovarian cancer” (PIP-0160-2022); the 39 participants represent all consecutively recruited women with an available serum aliquot and complete miRNA data. Participants were recruited in southern Spain from the Cádiz Health Area (Puerto Real University Hospital, PRUH) and the Seville Health Area (Virgen del Rocío University Hospital, VRUH). The Research Ethics Committee of Cádiz granted ethical approval (Code: ALGORITMOS, No. 118.22), and all procedures adhered to the Declaration of Helsinki [15]. Reporting follows the STARD 2015 statement [16]; a STARD checklist and flow diagram are provided as Supplementary Materials.

### 2.1. Recruitment and Patient Categorisation

Consecutive patients presenting with an adnexal mass and a surgical indication were recruited at PRUH or VRUH between June 2023 and November 2024 until the target of 20 patients with histopathologically confirmed ovarian cancer had been reached; a total of 90 patients were enrolled. Inclusion criteria were female sex (any age), an ovarian tumour on imaging, and a scheduled laparoscopy or laparotomy; exclusion criteria were a history of any previous neoplasia, chronic renal or hepatic disease, immunosuppressive treatment, or any surgery within the preceding 6 months. Histopathological examination identified 70 benign ovarian tumours and 20 ovarian cancers. Nine patients were excluded: three previously enrolled in the study (all benign), one receiving immunosuppressive therapy with methotrexate (benign), and five with a previous history of malignancy (four benign, one ovarian cancer), leaving 81 eligible patients (62 benign and 19 ovarian cancers). The final study cohort comprised the first 20 patients with benign tumours, selected according to the order of recruitment, together with all 19 eligible patients with ovarian cancer, i.e., 39 women; the full participant flow is shown in the STARD diagram (Supplementary Figure S8). All participants underwent surgery, and the reference standard was histopathology of the surgical specimen for every case, benign and malignant alike; no participant was classified on the basis of clinical–radiological follow-up alone. Three borderline (low-malignant-potential) tumours were grouped with the malignant tumours in the primary analysis, with a pre-specified sensitivity analysis excluding them, given their distinct biology. Malignant cases were staged according to the FIGO system [17]. One malignant participant had contributed a duplicate serum aliquot in the laboratory dataset; the redundant aliquot was removed at data cleaning so that each of the 39 participants contributed a single sample and the participant count was unaffected.

### 2.2. Blood Sampling, RNA Isolation and qPCR

Blood was collected on the day of surgery, during the pre-anaesthesia assessment, by peripheral venepuncture into three 9 mL VACUETTE serum gel separator tubes (Greiner Bio-One, Kremsmunster, Austria). After clotting for at least 30 min at room temperature, tubes were centrifuged at 3200× *g* (4000 rpm) for 7 min using a Consul 21 R centrifuge (Ortoalresa, Madrid, Spain); serum was immediately aliquoted into three 3 mL aliquots and stored at −80 °C until analysis (storage time 2–20 months). All samples were thawed only once, immediately before RNA extraction, avoiding repeated freeze–thaw cycles, and samples from both groups were processed and stored under identical pre-analytical conditions throughout the study. Serum quality was assessed for every sample by simultaneous measurement of the haemolysis (H-index), icteric (I-index) and lipaemic (L-index) indices on an Alinity c analyser (Abbott Laboratories, Chicago, IL, USA) using a multi-wavelength spectrophotometric method that corrects for spectral overlap among free haemoglobin, bilirubin and lipoproteins; all samples were within the laboratory’s predefined acceptance limits. As part of routine care, HE4 and CA 125 were measured by chemiluminescent microparticle immunoassay (CMIA) on an Alinity i analyser (Abbott). Total RNA was isolated from 200 uL serum using the miRNeasy Serum/Plasma Advanced Kit (Qiagen, Venlo, The Netherlands) with synthetic spike-ins (UniSp2, UniSp4, UniSp5) and MS2 carrier RNA (Roche, Basel, Switzerland). Reverse transcription used the miRCURY LNA RT Kit and qPCR the miRCURY LNA SYBR Green PCR Kit with the miRCURY LNA miRNA Serum/Plasma Focus PCR Panel (179 miRNAs; Qiagen) on a CFX Connect system (Bio-Rad, Hercules, CA, USA). To minimise inter-assay variability all samples were processed and analysed in a single analytical batch; inter-plate variability was monitored with UniSp3 and spike-in performance with UniSp2/UniSp4/UniSp5; no sample failed the spike-in or inter-plate quality-control criteria (0 of 40 aliquots). The study was conducted by two independent teams: the clinical investigators performed recruitment and histopathological confirmation, whereas the investigators who performed the qPCR miRNA analyses were blinded to the clinical and histopathological diagnosis. Cq values exceeding 35 (or undetermined up to 40 cycles) were considered undetectable; undetectable hsa-miR-200c-3p occurred in 0/20 benign and 4/19 (21%) malignant samples. Consistent with the acceptable serum-quality indices, an in silico haemolysis check based on the ΔCq (hsa-miR-23a-3p − hsa-miR-451a) index flagged only 3/39 samples above the risk threshold (>7), and none of the four non-amplifying malignant samples were haemolysed (index 2.3–4.5).

Reference-gene stability was assessed with RefFinder [18], which integrates geNorm, NormFinder, BestKeeper and the comparative ΔCt method. miR-103a-3p was the most stable endogenous control (median Cq 27.65 [26.19–28.95]). Relative quantification used the ^2−^ΔCq method (ΔCq = Cq_miRNA − median Cq_miR-103a-3p); values were log-transformed for analysis. Of the 179 panel miRNAs, 178 were screened for differential expression, miR-103a-3p being excluded as the reference gene. To confirm that findings were not an artefact of the reference gene, hsa-miR-200c-3p was additionally re-analysed under an alternative reference gene (miR-16-5p) and under global-mean and median normalisation. Discrimination was robust to the normalisation strategy (complete-case AUC 0.893 with miR-103a-3p, 0.903 with miR-16-5p, 0.890 under global-mean and 0.893 under median normalisation), whereas unnormalised raw Cq discriminated less well (AUC 0.770). The higher miR-103a-3p Cq in malignant samples (median 29.0 vs. 26.5; *p* = 0.001) paralleled a global reduction in circulating miRNA (per-sample mean panel Cq 31.2 vs. 30.1; *p* = 0.001) rather than specific dysregulation of the reference gene; multi-reference and haemolysis-controlled normalisation is recommended for the validation cohort.

### 2.3. Statistical Analysis

Normality was assessed with the Shapiro–Wilk test. Baseline variables were compared with the *t*-test or Mann–Whitney U test, and categorical variables were compared with the chi-squared or Fisher’s exact test. Differential expression across the 178 miRNAs was screened with the Mann–Whitney U test, and raw *p*-values were adjusted with the Benjamini–Hochberg FDR method [19]; significance was set at q < 0.05. hsa-miR-200c-3p was pre-specified as the primary candidate. Its fold-change is reported as the ratio of group medians of 2^−^ΔC^q^. Diagnostic performance was assessed by ROC analysis, reporting the AUC with bootstrap 95% confidence intervals (2000 resamples), the Youden cut-off, sensitivity, specificity and predictive values. Non-amplifying hsa-miR-200c-3p results were handled in two pre-specified ways applied identically across ROC and regression analyses: a complete-case analysis restricted to participants with a detectable marker (*n* = 35), and an all-case analysis imputing non-amplifying samples as low expression (*n* = 39); both are reported side by side and the all-case analysis is considered at least as clinically informative. A further sensitivity analysis excluded borderline tumours. AUCs of hsa-miR-200c-3p, CA 125, HE4 and age were compared on common complete cases (DeLong method [20]). Because of quasi-separation with only 19 events, multivariable models were fitted with Firth penalised-likelihood regression [21] using penalised likelihood-ratio tests, reporting standardised coefficients, 95% confidence intervals and events-per-variable; conventional maximum-likelihood coefficients are unstable and are provided only in Supplementary Table S3. Apparent AUCs were internally validated by bootstrap optimism correction. Supporting structure was explored with PCA and LASSO logistic regression (standardised predictors; 5-fold cross-validation; AUC-optimal lambda); these analyses, and all multivariable models, are exploratory and hypothesis-generating, and were pre-specified independently of the outcome for hsa-miR-200c-3p; TRIPOD guidance [22] informs this exploratory component. Analyses were performed in Python 3.10.12 (NumPy 1.26.4, pandas 2.3.3, SciPy 1.15.3, scikit-learn 1.7.2; figures produced with Matplotlib 3.10.9); the full code is openly available (see Data Availability).

## 3. Results

The cohort comprised 39 participants: 20 benign and 19 malignant tumours (Table 1). Patients with malignant tumours were significantly older (62.7 ± 12.1 vs. 42.7 ± 12.3 years; *p* < 0.001) and more frequently postmenopausal (84.2% vs. 20.0%; *p* < 0.001) and hypertensive (42.1% vs. 5.0%; *p* = 0.008). Serum CA 125 and HE4 were markedly higher in the malignant group (both *p* < 0.001). Benign tumours comprised 13 (65.0%) functional cysts or endometriomas, 6 (30.0%) mature cystic teratomas or struma ovarii, and 1 (5.0%) serous cystadenoma. Malignant tumours comprised 13 high-grade serous, 1 low-grade serous (FIGO IC1) and 2 invasive mucinous carcinomas, together with 3 borderline tumours (2 mucinous, 1 serous); grades were assigned from pathology reports where available. FIGO stage was I in five (26.3%), III in seven (36.8%) and IV in seven (36.8%); no FIGO Stage II cases were present in this cohort.

Of the 178 miRNAs analysed, 71 showed nominal differences (*p* < 0.05) and 38 remained significant after FDR correction (q < 0.05; Supplementary Table S1). The leading miRNAs are shown in Table 2; hsa-miR-200c-3p and hsa-miR-141-3p—both miR-200 family members—ranked first and second, supporting the biological relevance of this family. Individual expression profiles are shown in Supplementary Figures S1–S7.

**Figure 1:**
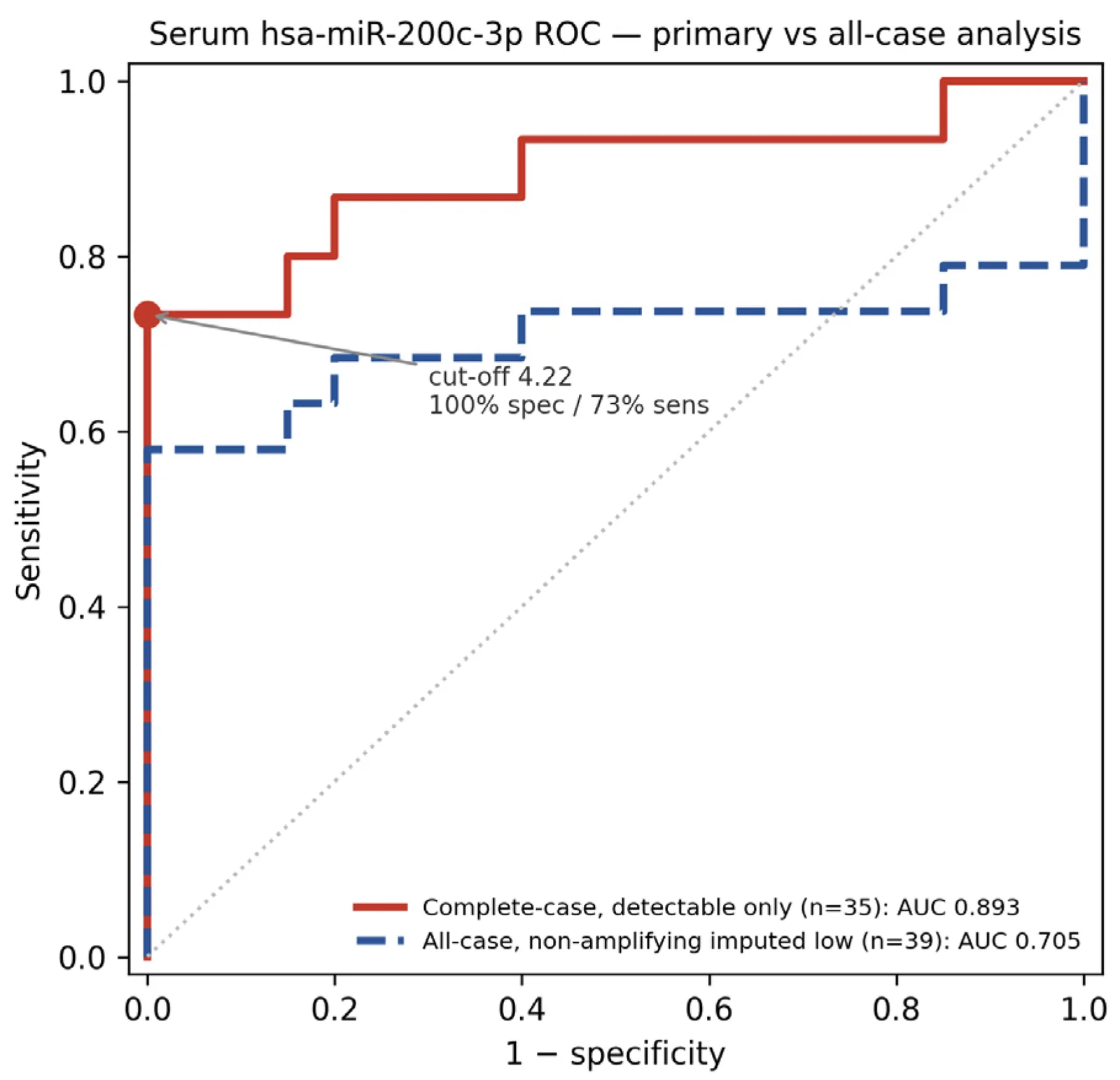
Receiver operating characteristic (ROC) curves for serum hsa-miR-200c-3p distinguishing benign from malignant ovarian tumours. The solid curve is the primary complete-case analysis (*n* = 35; 20 benign, 15 malignant), which excludes four high-grade serous carcinomas with non-amplification of hsa-miR-200c-3p (AUC 0.893; a relative-expression cut-off of 4.22 gave 100% specificity and 73% sensitivity). The dashed curve is the all-case analysis with the four non-amplifying samples imputed as low expression (*n* = 39; AUC 0.705). The dotted diagonal represents no discrimination (AUC = 0.5).

Serum hsa-miR-200c-3p was up-regulated in malignant tumours (17.6-fold by the ratio of group medians; q = 0.014). In the primary complete-case analysis, it discriminated benign tumours from malignant tumours with an AUC of 0.893 (95% CI 0.75–0.99); at a relative-expression cut-off of 4.22, specificity was 100% with a sensitivity of 73% (Table 3, Figure 1). The finding was robust to the normalisation strategy: AUC 0.890 under global-mean and 0.893 under median normalisation, and 0.903 with an alternative reference gene (miR-16-5p); the cut-off, sensitivity and specificity for each normalisation strategy are reported in Supplementary Table S2, confirming it was not an artefact of the endogenous reference gene, whose Cq itself differed between groups (miR-103a-3p median Cq 29.0 in malignant vs. 26.5 in benign; *p* = 0.001) in parallel with globally lower circulating miRNA levels in the malignant group (higher per-sample mean Cq, 31.0 vs. 29.8; *p* = 0.001), consistent with reduced overall serum miRNA abundance in cancer rather than specific dysregulation of the reference gene.

Importantly, four high-grade serous carcinomas showed no amplification of hsa-miR-200c-3p (undetermined Cq at any threshold up to 40). These were all advanced-stage tumours (FIGO III–IV) with markedly elevated conventional markers (CA 125 182–3569 U/mL; HE4 225–19, 940 pM)—that is, tumours readily detected by CA 125/HE4. In the sensitivity analysis imputing these as low expression, the AUC fell to 0.705 (0.50–0.88), whereas excluding borderline tumours increased it to 0.971 (0.91–1.00). The complete-case estimate should therefore be regarded as optimistic, and a technical (low-abundance) contribution to the non-amplification cannot be excluded.

As a single discriminator, hsa-miR-200c-3p (AUC 0.893, 95% CI 0.76–0.99) did not differ significantly from HE4 (0.913), CA 125 (0.867) or age (0.860); all pairwise comparisons yielded *p* > 0.5 (Table 4). Under Firth penalised regression, which accounts for quasi-separation, hsa-miR-200c-3p retained an association with malignancy beyond age (OR per SD 9.7; penalised-LR *p* = 0.001), CA 125 (*p* = 0.003) and HE4 (OR per SD 7.9, 95% CI 1.5–42.3; *p* = 0.010); however, confidence intervals were wide and, in the full model including age, CA 125 and HE4, the events-per-variable ratio was only 3.8. These analyses are therefore exploratory and hypothesis-generating and are not interpreted as demonstrating clinically decisive incremental value (full model specifications, standardised coefficients, 95% confidence intervals, events-per-variable, and apparent and bootstrap optimism-corrected AUCs are provided in Supplementary Table S3). The bootstrap optimism-corrected AUC of hsa-miR-200c-3p alone was 0.888, indicating minimal overfitting for a single predictor.

**Figure 2:**
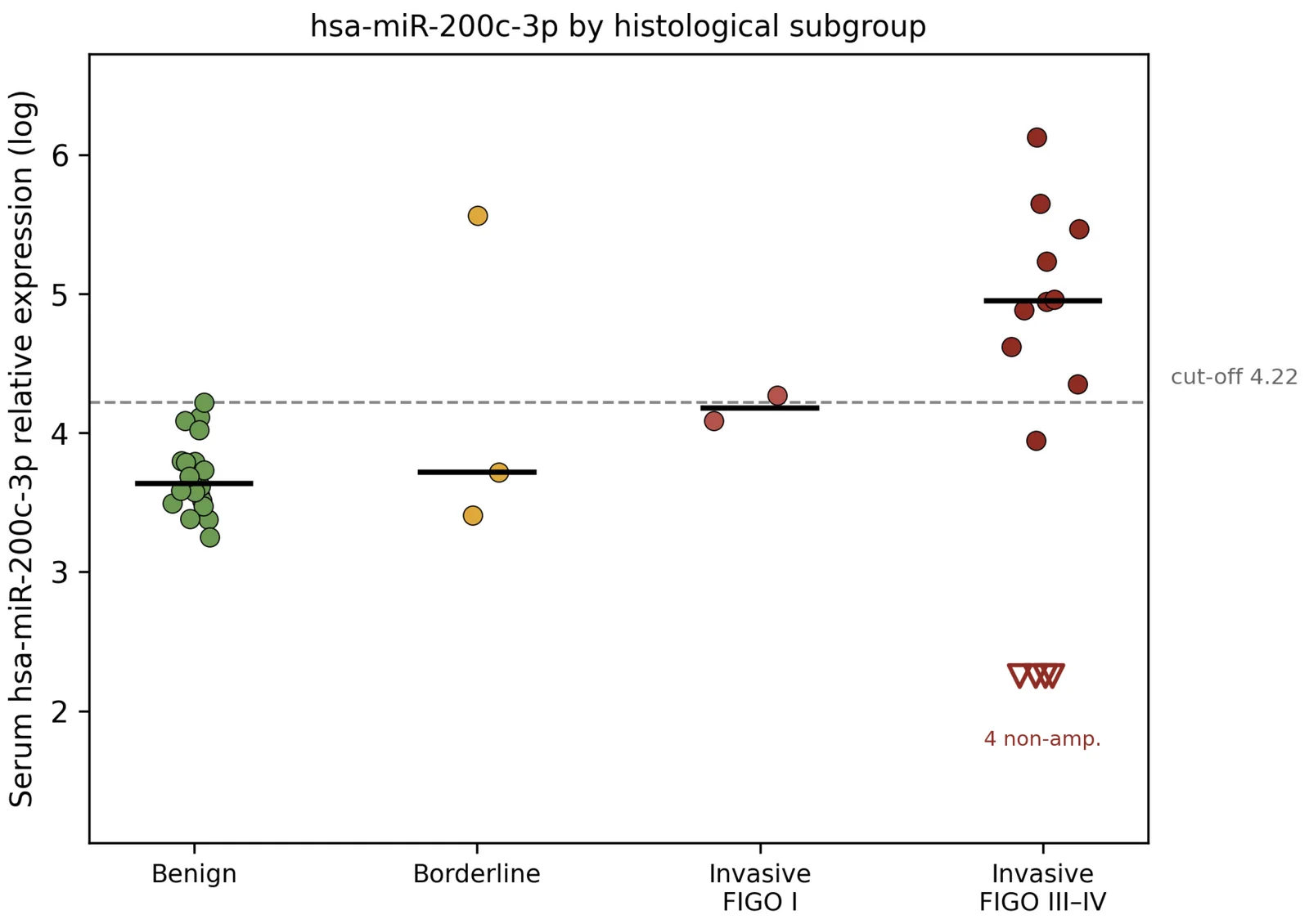
Individual serum hsa-miR-200c-3p relative expression by histological subgroup (benign, borderline, invasive FIGO I, and invasive FIGO III–IV). Horizontal bars denote group medians and the dashed line the 4.22 cut-off. The four non-amplifying malignant cases (all high-grade serous carcinomas, FIGO III–IV) are shown as open downward triangles at the foot of the advanced-stage column. Colours denote the histological subgroups indicated on the x-axis (for visual distinction only). Open triangles mark the four non-amplifying high-grade serous carcinomas, plotted at the foot of the panel. The dashed line indicates the 4.22 relative-expression cut-off; horizontal bars are group medians.

Serum hsa-miR-200c-3p did not differ between serous and mucinous carcinomas (4.69 ± 0.68 vs. 4.92 ± 1.11; *p* = 0.75), suggesting comparable performance across the major epithelial subtypes; a non-significant trend toward higher expression with stage was observed (FIGO I 4.21 ± 0.83; III 5.11 ± 0.58; IV 4.96 ± 0.72; ANOVA *p* = 0.16) (Figure 2). In the exploratory analyses, principal-component analysis (on standardised expression) separated the groups along the first two components (PC1 42.6% and PC2 13.5% of variance). LASSO-penalised logistic regression (standardised predictors; 5-fold cross-validation; penalty chosen by the AUC-optimal rule, lambda = 1.37), restricted to the miRNAs detectable in all participants, selected an unstable subset that did not include hsa-miR-200c-3p (ineligible owing to its non-amplification in four samples). Both analyses are exploratory and illustrate the instability of data-driven feature selection at this sample size, consistent with the complex relationship between the miRNA-biogenesis machinery and ovarian cancer phenotypes [23] (see Section 4).

## 4. Discussion

In this exploratory study, hsa-miR-200c-3p was up-regulated in malignant ovarian tumours. Its apparent discrimination was good in the complete-case analysis (AUC 0.893) but fell to 0.705 when the four non-amplifying high-grade serous carcinomas were included, so the complete-case estimate should be read alongside the all-case analysis rather than in isolation. Robust differential expression was nonetheless evident: 38 miRNAs remained significant after FDR correction, with hsa-miR-200c-3p and hsa-miR-141-3p—both miR-200 family members—as the leading signals, consistent with evidence implicating the miR-200 family in epithelial OC.

While miR-200 family members, including miR-200c, have traditionally been characterised as suppressors of epithelial–mesenchymal transition through targeting of ZEB1/ZEB2 and thus as inhibitors of metastasis in many cancers [7], the context of OC is distinct: the miR-200 family is consistently over-expressed in malignant ovarian tissue, where it may promote tumour-cell growth, facilitate cortical inclusion cyst formation and enable collective peritoneal dissemination, thereby supporting malignant phenotypes [7,13]. Induced miR-200 expression may be an early event in the transformation of ovarian epithelial cells [24], and miR-200c-3p has been shown to drive high-grade serous OC progression by suppressing the tumour-suppressor gene DLC1 [25]. Notably, the same molecule can also act in a tumour-suppressive, context-dependent manner: Anastasiadou et al. reported that miR-200c-3p down-regulates β-catenin and c-Myc and counteracts PD-L1 induction in epithelial ovarian cancer [26]. This dual, context-dependent behaviour underlines that circulating miR-200c-3p should be interpreted as a disease-associated marker rather than a simple oncogene, and the concordant up-regulation of hsa-miR-141-3p supports a family-level effect.

Our findings are concordant with a growing body of evidence on circulating miR-200 family members in ovarian cancer. Reported AUCs for serum or plasma miR-200c-3p alone typically range from approximately 0.78 to 0.85 in comparisons of epithelial ovarian cancer with benign or control samples, rising above 0.90 when the marker is combined with other miR-200 members or with CA 125 [27,28], and a pooled meta-analysis has confirmed its diagnostic value across independent cohorts [29]; our primary AUC of 0.893 sits at the upper end of the single-marker range. The concordant up-regulation of hsa-miR-141-3p in our data mirrors panels in which miR-200c, miR-141 and miR-205 are jointly elevated in epithelial ovarian cancer, an effect attributed to promoter hypomethylation and to the retained epithelial identity of high-grade serous tumours, which abundantly express miR-200/141 to maintain the epithelial phenotype through ZEB1/ZEB2 repression [27,28]. Finally, the non-amplification of hsa-miR-200c-3p in four high-grade serous carcinomas is consistent with reports of matrix-dependent detection of miR-200 members, in which discrimination is achievable in some biofluids but not others despite tissue over-expression [30], reinforcing that pre-analytical and low-abundance factors, rather than absence of the biomarker, may underlie these cases.

Two findings temper the clinical interpretation and were addressed transparently. First, four high-grade serous carcinomas showed no amplification of serum hsa-miR-200c-3p; because these were excluded from the primary complete-case analysis, the AUC of 0.893 is optimistic, and imputing them as low expression reduced it to 0.705. These four tumours had very high CA 125 and HE4 and were not haemolysed, so their non-amplification most plausibly reflects genuinely low circulating target abundance rather than a pre-analytical artefact; nonetheless, a marker non-amplified in approximately one-fifth of malignant cases requires dedicated technical and clinical validation, and any combined diagnostic strategy would need to be demonstrated in the full cohort. Second, the malignant group was substantially older and more frequently postmenopausal, and both age and the established markers CA 125 and HE4 discriminated as well as hsa-miR-200c-3p, with HE4 numerically best; hsa-miR-200c-3p is therefore not superior to existing markers. Although it retained an association beyond age, CA 125 and HE4 under penalised regression, the wide confidence intervals, absence of formal multiplicity adjustment and possible residual confounding mean any incremental value should be regarded as exploratory rather than clinically decisive.

The supporting exploratory analyses should be interpreted with caution. LASSO-penalised regression applied to the miRNAs detectable in all participants selected an unstable subset; because hsa-miR-200c-3p has missing values by virtue of non-amplification, it was not eligible for that particular selection. The instability of data-driven selection at this sample size, and its sensitivity to the correction of only a few cases relative to an earlier, pre-correction internal analysis of the same dataset (before the data-cleaning steps, namely removal of the redundant serum aliquot and reclassification of two cases), motivated our decision to pre-specify hsa-miR-200c-3p on biological grounds rather than to rely on the selection itself.

From a translational perspective, a single-target serum assay would be cost-effective, technically straightforward and readily adaptable to routine laboratory workflows. Serum hsa-miR-200c-3p did not differ between serous and mucinous carcinomas, suggesting consistent behaviour across the major epithelial subtypes. In principle, a high-specificity operating point maximises positive predictive value at a given disease prevalence; however, because this cut-off was both derived and evaluated in the same small cohort, it is not ready for clinical or surgical-triage use and would require prospective, independent validation before any such application could be considered. Rather than replacing imaging or established markers, hsa-miR-200c-3p is best positioned as a complement to CA 125, HE4 and the ROMA algorithm within multimodal preoperative triage [1,2].

This study has limitations. The sample is small (*n* = 39) and single-project, precluding external validation and stratified analyses of rare subtypes; the malignant group was significantly older, so residual confounding by age and menopausal status cannot be fully excluded; four high-grade serous carcinomas did not amplify, biasing the complete-case estimate; three borderline tumours were included in the malignant group (a sensitivity analysis excluding them is reported); a single endogenous reference gene was used, and although the finding was robust to alternative normalisation, MIQE-compliant multi-reference and haemolysis-controlled normalisation is recommended for confirmatory work; and the cut-off and its performance were derived and evaluated in the same small cohort, so the operating point is not ready for surgical triage. Strengths include histopathological reference-standard confirmation in all participants, the clinically relevant comparator (benign adnexal masses rather than healthy controls), FDR control, formal sensitivity and normalisation-robustness analyses, an in silico haemolysis assessment, comparison with and adjustment for established markers, penalised-regression and bootstrap internal validation, and stringent spike-in-based quality control.

## 5. Conclusions

Serum hsa-miR-200c-3p is a preliminary candidate for the preoperative discrimination of benign from malignant adnexal masses: it is up-regulated in malignancy but is not superior to CA 125 or HE4, and it is non-amplified in a clinically relevant subset of high-grade serous carcinomas, so that the AUC falls from 0.893 (complete-case) to 0.705 when these are included. Any incremental value beyond age and established markers is modest and exploratory, and residual confounding by age and menopausal status cannot be excluded. These hypothesis-generating findings require a prospectively recruited, adequately powered, age-matched independent validation cohort using pre-specified assays and analysis plans before any diagnostic use can be considered.

## Figures and Tables

**Table 1:** Baseline characteristics (benign *n* = 20; malignant *n* = 19).

Characteristic	Benign (*n* = 20)	Malignant (*n* = 19)	*p*-Value
Age, mean ± SD (years)	42.7 ± 12.3	62.7 ± 12.1	<0.001
Postmenopausal, *n* (%)	4 (20.0)	16 (84.2)	<0.001
Hypertension, *n* (%)	1 (5.0)	8 (42.1)	0.008
CA 125 (U/mL), median [IQR]	55.9 [16.9–138.3]	710.5 [172.6–2169.8]	<0.001
HE4 (pmol/L), median [IQR]	36.2 [30.0–53.5]	240.0 [77.3–779.4]	<0.001
Functional cyst/endometrioma, *n* (%)	13 (65.0)	—	—
Mature teratoma/struma, *n* (%)	6 (30.0)	—	—
Serous cystadenoma, *n* (%)	1 (5.0)	—	—
High-grade serous carcinoma, *n*	—	13	—
Low-grade serous carcinoma, *n*	—	1	—
Invasive mucinous carcinoma, *n*	—	2	—
Borderline tumour (2 mucinous, 1 serous), *n*	—	3	—
FIGO I/III/IV, *n*	—	5/7/7	—

Differences significant for age, menopausal status, hypertension, CA 125 and HE4. No FIGO Stage II cases were present. FIGO, International Federation of Gynaecology and Obstetrics.

**Table 2:** Leading differentially expressed serum miRNAs (FDR-corrected; full list in Supplementary Table S1).

miRNA	Direction	Fold-Change	*p* (MWU)	q (FDR)	AUC
hsa-miR-200c-3p	up	17.7×	0.0001	0.013	0.893
hsa-miR-141-3p	up	8.8×	0.0002	0.013	0.884
hsa-miR-125b-5p	up	2.9×	0.0003	0.015	0.850
hsa-miR-10b-5p	up	2.7×	0.0004	0.015	0.841
hsa-miR-99a-5p	up	2.8×	0.0004	0.015	0.841
hsa-miR-100-5p	up	2.6×	0.0006	0.017	0.838
hsa-miR-93-3p	up	2.8×	0.0008	0.020	0.864

Of 178 miRNAs, 71 had *p* < 0.05 and 38 survived FDR correction. hsa-miR-200c-3p and hsa-miR-141-3p belong to the miR-200 family.

**Table 3:** Diagnostic performance of serum hsa-miR-200c-3p across scenarios.

Scenario	*n* (Benign/Malignant)	AUC	95% CI
Primary, detectable only	35 (20/15)	0.893	0.76–0.99
All-case, non-amplifying imputed low	39 (20/19)	0.705	0.51–0.88
Excluding borderline (detectable)	32 (20/12)	0.971	0.91–1.00
Excluding borderline + imputed	36 (20/16)	0.728	0.49–0.93
miR-16-5p reference (detectable)	35 (20/15)	0.903	0.75–1.00
Global-mean normalisation (detectable)	35 (20/15)	0.890	0.74–0.99
Median normalisation (detectable)	35 (20/15)	0.893	0.74–0.99

Four high-grade serous carcinomas showed no amplification of hsa-miR-200c-3p. At the Youden cut-off (4.22) in the primary analysis, specificity was 100% and sensitivity 73%. AUCs are reported with bootstrap 95% confidence intervals; group denominators are benign/malignant. The reference-gene, miR-16-5p, global-mean and median rows share the same 35 complete-case participants; the median-normalisation AUC coincides numerically with the primary value but corresponds to a different operating point (Supplementary Table S2).

**Table 4:** Single-marker discrimination and comparison with hsa-miR-200c-3p (n = 35).

Marker	AUC [95% CI] (*n* = 35)	Δ vs. hsa-miR-200c-3p
hsa-miR-200c-3p	0.893 [0.76–0.99]	reference
HE4	0.913 [0.79–1.00]	+0.020 (n.s.)
CA 125	0.867 [0.74–0.98]	−0.027 (n.s.)
Age	0.860 [0.72–0.97]	−0.033 (n.s.)

Δ, difference in AUC relative to hsa-miR-200c-3p (positive values indicate a higher AUC than hsa-miR-200c-3p), estimated on the 35 common complete cases; n.s., not significant — pairwise comparison of AUCs by the DeLong test yielded *p* > 0.5. AUC, area under the receiver operating characteristic curve; CI, confidence interval. No pairwise difference was significant. In multivariable models, hsa-miR-200c-3p added discrimination beyond age (LR *p* = 0.0003), CA 125 (LR *p* = 0.011) and HE4 (LR *p* = 0.018).

## Data Availability

The individual-level Cq data for the 178 miRNAs are provided as Supplementary Dataset S1. The full analysis code (differential expression, ROC with bootstrap confidence intervals, Firth regression, PCA and LASSO) is openly available at Zenodo (https://doi.org/10.5281/zenodo.21650600). Further inquiries can be directed to the corresponding authors.

## Supplementary Materials

The following supporting information can be downloaded at https://www.mdpi.com/article/10.3390/diagnostics16172718/s1. Table S1: Complete list of the 38 FDR-significant serum miRNAs. Table S2: Diagnostic performance of serum hsa-miR-200c-3p under alternative normalisation strategies (complete-case, *n* = 35; 20 benign, 15 malignant). Table S3: Firth penalized-likelihood logistic regression models (standardized predictors; complete-case *n* = 35, 15 events). Figure S1–S7: Individual serum expression profiles of the differentially expressed miRNAs; Figure S8: STARD participant-flow diagram. Dataset S1: Individual-level raw quantification-cycle (Cq) values for the 178 serum miRNAs.
